# Strategies to Increase Uptake of Parent Education Programs in Preschool and School Settings to Improve Child Outcomes: A Delphi Study

**DOI:** 10.3390/ijerph18073524

**Published:** 2021-03-29

**Authors:** Wan Hua Sim, John W. Toumbourou, Elizabeth M. Clancy, Elizabeth M. Westrupp, Michelle L. Benstead, Marie B. H. Yap

**Affiliations:** 1 Turner Institute for Brain and Mental Health, School of Psychological Sciences, Monash University, Clayton 3800, Australia; wan.sim@monash.edu; 2 Centre for Social and Early Emotional Development, School of Psychology, Deakin University, Geelong 3220, Australia; john.toumbourou@deakin.edu.au (J.W.T.); elizabeth.clancy@deakin.edu.au (E.M.C.); elizabeth.westrupp@deakin.edu.au (E.M.W.); michelle.benstead@deakin.edu.au (M.L.B.); 3 Melbourne School of Population and Global Health, University of Melbourne, Carlton 3053, Australia

**Keywords:** family–school, family engagement, home-school, intervention, mental health, parent engagement, parent involvement, partnership, prevention, recruitment

## Abstract

Parent education programs, offered via family–school partnerships, offer an effective means for promoting the mental health and educational functioning of children and adolescents at a whole-school level. However, these programs often have a low uptake. This study aimed to identify strategies for increasing the uptake of parent education programs within preschool and school settings. A three-round Delphi procedure was employed to obtain expert consensus on strategies that are important and feasible in educational settings. First, thirty experts rated statements identified from the literature and a stakeholder forum. Next, experts re-appraised statements, including new statements generated from the first round. Ninety statements were endorsed by ≥80% of the experts. Primary themes include strategies for program selection; strategies for increasing the accessibility of programs and the understanding of educational staff on parent engagement and child mental health; strategies for program development, promotion and delivery; as well as strategies for increasing parent and community engagement. This study offers a set of consensus strategies for improving the uptake of parent education programs within family–school partnership.

## 1. Introduction

Over the course of the schooling years (typically ages 4–17), young people create and consolidate patterns of cognitions and behaviours that affect their immediate and long-term wellbeing. The school years are also a period of the life-course where young people are progressively at increased risk for mental health problems and educational disengagement [1,2,3], which in turn can lead to a cascade of long-term negative outcomes [4,5,6]. A growing body of research suggests that children who are mentally healthy learn better, and, reciprocally, adults who are more educated enjoy healthier, more productive and longer lives [7,8,9]. Given that both risk and protective factors for mental health run through the early childhood and school years [10,11], it is prudent that prevention and response efforts are strengthened in the primary settings where young people are socialised—families and schools. 

While early learning centres and schools have conventionally been championed as conduits for academic, government and nongovernment health and social services to access and provide support to children and families, the home-school mesosystem may be the most compelling avenue for enhancing outcomes for children and adolescents. The bulk of existing research on family–school partnerships has focused on how educators can work with parents and other primary caregivers/guardians (herein collectively referred to as “parents”) to improve child educational and/or behavioural outcomes. In the education literature, parents’ engagement (also frequently termed “parent involvement” [12]) in their child’s home-based learning and school activities is associated with a child’s school readiness, school attendance, academic achievement, social-emotional skills and prosocial behaviour [13,14,15,16,17]. Research shows that parent engagement may improve child educational and psychosocial adjustment outcomes by increasing parents’ social capital and social control [18], children’s parent-oriented motivation [19] and school engagement [17,20]. Although the benefits of parent engagement in education are clear, there are several challenges to building effective parent engagement and family–school partnerships.

Common barriers to parent engagement in education include constraints of time and resources, parents’ low self-efficacy or confidence in their ability to support their child’s learning, fear of retaliation and language- and culture-related barriers [21,22]. For educators, barriers relate to time constraints and a lack of training and skills for working effectively with parents to facilitate a child’s learning [22]. In addition, parent–teacher factors such as differences in values, beliefs and expectations about what should be done and what is helpful and lack of mutual trust and understanding have also been cited as barriers to successful family–school partnerships [21,23]. While several comprehensive frameworks for measuring parent and family engagement have emerged in recent years [24,25,26,27], recommended best practices and processes have not yet been systematically and empirically evaluated for their effects on child and adolescent outcomes. 

Parental factors associated with child mental health outcomes overlap with the parental factors that influence child educational outcomes [28,29,30]. Thus, there is reason to believe that parent education programs that address these common factors are also likely to improve child mental health, in turn leading to beneficial effects on child educational outcomes. Parent education programs are interventions that systematically assist parents to gain the knowledge and skills required to mediate or extend the intervention with their child in various care-giving contexts, with the aim of promoting their child’s development or other desired outcomes [31]. While there is a paucity of research examining which family–school partnership strategies are effective for improving child mental health outcomes, there is considerable evidence that parent education programs are effective in improving parenting skills and practices and in reducing child internalising problems such as anxiety and depression [32,33]. Therefore, studies on parent education programs that have been delivered in educational settings might provide some insights into the factors that support program implementation in educational settings and benefit child mental health and educational outcomes. 

A review by Mendez et al. [34] on parent involvement in school-based mental health interventions suggests that the majority of the interventions have focused on enhancing parenting skills to prevent the onset or escalation of maladaptive behaviors in children, with some demonstrating concomitant positive effects on mental health outcomes. A more recent rapid review by Clancy et al. [35] showed an ongoing dearth of research on the impact of family–school partnerships on child mental health and wellbeing and a lack of robust evidence for existing practices in building and maintaining family–school partnership for enhancing mental health and wellbeing in children and adolescents. Nonetheless, the review identified several partnership strategies with emerging evidence for improving mental health in children and adolescents through family service delivery in educational settings. Partnership strategies supported by at least two well-designed studies demonstrating evidence of effects included (i) preschool services referring vulnerable families to receive home visiting, (ii) schools inviting families into facilitated sessions where teachers are involved, (iii) schools inviting families and hosting sessions with teachers’ participation, (iv) implementing a manualised program for children with high anxiety that can be delivered by school staff with parent education materials provided, and (v) including student curricula and parent interventions that are implemented by school staff. Of note, these partnership strategies have typically been implemented and evaluated as part of a larger intervention program that includes activities for parents. 

Consistent with Garbacz et al. [26] who emphasised the importance of the school climate and staff–parent interactions, several of the evidence-based programs (e.g., Strengthening Families, Families and Schools Together) identified from Clancy et al.’s review [35] comprised activities to train and prepare staff for family engagement work. A recent meta-analysis [36] shows that only interventions with relational components such as collaboration or school-to-home communication showed positive impact on child mental health outcomes (operationalised as internalising behaviour, self-esteem or self-worth), with pooled effect sizes δ = 0.29 for collaboration and δ = 0.73 for school-to-home communication. However, very few studies reported data on mental health outcomes that could be included in the meta-analysis, and broad definitions were used to define mental health outcomes and the various components of family–school partnerships. Taken together, these findings nonetheless suggest that family and parent education programs may be important elements that contribute to family–school partnerships that seek to improve child and adolescent mental health outcomes. 

The uptake of parent education programs in educational settings is influenced by factors associated with parents, as well as those associated with educators (representing schools) [37,38]. In the space of preventative interventions that address family and individual factors, research suggests that between 30 to 85% of parents identified as eligible for a parent education program actually engaged with and completed the program, and about half of enrolled parents dropped out prior to completing the program [39,40]. Even for programs with demonstrated efficacy in initial trials, replicating similar effects in real world settings has been challenging due to difficulties in recruitment and retention of parents [41]. Systematic reviews [39,40] have shown that there are no to small associations between parental socio-economic status and parental uptake of parent education programs. More importantly, there is currently little evidence in the academic literature on effective, actionable strategies that educators, researchers and practitioners can use to increase the uptake of parent education programs in educational settings with the aim of supporting mental health and wellbeing in children and adolescents. Therefore, it is imperative to prioritise investigations into implementation strategies to increase the uptake of parent education programs in educational settings. 

As the main source of referrers to parent education programs in educational settings, school leaders and educators are often the gatekeepers of access to parents and therefore play an important role in influencing the uptake of family and parent education programs in schools. In light of limited funding, the tension of choosing between mental health and other priorities has prevented some schools from allocating resources to family and parent education programs. Factors related to a school’s emphasis on prevention, school staff’s beliefs and attitudes about an intervention, leadership style and behaviour of the school principal and the personal characteristics of implementers (e.g., enthusiasm, self-efficacy) have been reported to either facilitate or impede implementation [42]. Even when mental health is prioritised in schools, schools face barriers related to parent attitudes and engagement, student attitudes and access to specialists and external agencies [41,43]. 

To address gaps in the literature, the present study aimed to develop guidance for educators, program developers and facilitators and service providers on actions that each stakeholder can take to increase the uptake of parent education programs in educational settings. Specifically, to uncover strategies that would be sensitive to the climate of family–school engagement in Australia, the study employed the Delphi method to facilitate expert consensus on strategies that are both important and feasible for increasing the uptake of parent education programs in early learning and school settings in Australia. 

## 2. Materials and Methods

### 2.1. Study Design

The Delphi method seeks to obtain insights from subject experts about an issue, assess the extent of agreement and establish a convergence of opinion on an issue through an iterative process [44]. This method has been used extensively in health sciences education, as well as medical and mental health research, particularly for developing recommendations or guidelines for service planning and delivery and the development of curriculum, professional training, instruments and interventions [44,45]. The aim of a Delphi study is to achieve expert consensus rather than generalisability of the results; therefore, statistical power is less relevant for determining sample size [46]. Nonetheless, a simulation study shows that consensus can be reliably achieved with a sample of 23 suitably qualified panelists [47]. For the present study, a Delphi method that comprises three survey rounds was employed. This provides a systematic way for people with relevant expertise to obtain, share, revise or confirm their opinions in the context of a less robust evidence base around programs and strategies for improving parental engagement and uptake of programs in educational settings. 

### 2.2. Panel Formation

Purposive and snowballing sampling techniques were used given that the Delphi method requires participants with specific expertise. There is value in having representation from diverse members with relevant expertise [45], thus, the study recruited expert participants with a minimum of five years’ experience across research, education, or service provision roles in family–school partnerships and/or engagement of families in programs involving parenting, child wellbeing and educational outcomes. These participant groups were selected in the sample frame to represent the perspectives of the major sources of influence on the development, promotion and implementation of parent education programs in early learning and school settings. 

Potential expert participants were first identified through the attendance list of a workshop conducted by authors J.W.T., M.B.H.Y., E.M.C., E.M.W. and M.L.B. in Melbourne as part of a forum for stakeholders on family–school partnerships, co-hosted with the Australian Research Alliance for Children and Youth (ARACY). ARACY is a not-for-profit organisation that aims to improve the lives of children and young people by developing evidence-based solutions through partnerships and collaborations with educators, researchers, service providers and policymakers across Australia. Participants who consented to being contacted after the workshop (*n* = 30) were invited by email in June 2020 to take part in the Delphi study. Of these email invitations, five of the emails bounced or had their email accounts marked by the recipient server as having been disabled. Hence only 25 email invitations were successfully delivered to the prospective participants. In addition, generic invitations to participate in the study were sent to members of the ARACY’s parent engagement network, consisting of researchers, educators, parents, policy-makers and others interested in parent engagement in children’s learning. Further, professional contacts (*n* = 5) nominated by ARACY members were also invited to participate. It was expected that having researchers and educators on the panel would increase the likelihood that the strategies developed will conform to the experts’ current understandings of the best available evidence and recommended practices. As service providers are often in the frontline of engagement work with parents in educational settings and therefore would have the expertise to determine what strategies are likely to be important or effective, experienced service providers were also invited to be on the panel. The study was approved by the Monash University Human Research Ethics Committee.

### 2.3. Survey Development

The content for the survey items in Round 1 was developed based on the principles and potential strategies identified from (a) a secondary search of the abovementioned rapid review of interventions delivered/implemented in school settings with the primary aim of preventing child mental health problems [35]; (b) a workshop with stakeholders at a forum on family–school partnerships; and (c) an updated search of grey literature (e.g., materials and research outside traditional academic publishing, such as policy documents and reports) three months prior to the commencement of data collection. Details of these sources are elaborated next.

Drawing on an existing rapid review of interventions delivered/implemented in school settings with the primary aim of preventing child mental health problems (see [35]), W.H.S. performed a systematic search of the reviewed publications to determine principles and strategies available in the academic literature for improving parent engagement and uptake of programs in preschool and school settings. To further identify concepts and strategies that may be important for enhancing family–school partnership and in turn increase the uptake of parent programs, W.H.S. and a research assistant also reviewed notes recorded at a workshop conducted as part of the abovementioned family–school partnership forum in August 2019. Finally, a research assistant performed a search of the grey literature (e.g., policy documents, reports and newsletters from government agencies and philanthropic groups) to locate recommended practices for increasing program participation and engagement. Through this process, possible strategies identified were first written into statements by the same research assistant. They were then reviewed and revised by W.H.S. and M.B.H.Y. to ensure that each was clear and unique for rating in the first survey round. 

### 2.4. Survey Administration

The surveys were administered over three successive rounds using an online survey software program (Qualtrics). The panel was asked to provide two ratings for each item in the survey: one on its importance and another on its feasibility for increasing the uptake of parent education programs in educational settings. In line with previous Delphi consensus studies [48,49], five-point scales were used (1 = Essential/Very feasible, 2 = Important/Feasible, 3 = Don’t know/Depends, 4 = Unimportant/Not feasible, and 5 = Should not be included). The panelists had up to six weeks to complete the Round 1 survey (including time during a school winter break in Australia), six weeks to complete the Round 2 survey (including a school spring break) and two weeks to complete the Round 3 survey. Non-responders were sent up to three email reminders for each round. In this Delphi study, only experts who completed the survey in full in Round 1 were contacted for subsequent rounds. It should be noted that data collection took place between June and November 2020 during the COVID-19 pandemic, where in Australia, physical distancing measures were enforced, and most people were encouraged to work and study from home. 

The Round 1 survey comprised item-statements created from the process described before. Survey responses collected from Round 1 were then analysed to determine which statements were endorsed by panel members as “important” or “essential” and “feasible” or “very feasible” for increasing the uptake of parent education programs. Statements that did not attain clear consensus (below 80% consensus) in Round 1 were presented for re-rating in the Round 2 survey. In the Round 1 survey, panelists were also given the opportunity to provide feedback on the statements or suggest new statements for consideration. New ideas from the panel were then drafted into new item-statements by W.H.S. and reviewed by M.B.H.Y. before being incorporated into the Round 2 survey. Consequently, the Round 2 survey consisted of (1) items that did not achieve clear consensus in Round 1 and needed to be re-rated and (2) new items to be rated for the first time. Finally, new items in Round 2 that did not establish clear consensus were presented for re-rating in the third (final) round. In Round 3, the survey was made up of items from Round 2 that required re-rating due to an inadequate level of consensus; no new items were introduced. In Rounds 2 and 3, the panel was also provided with a document that thematically grouped the items endorsed by at least 80% of the panel in the previous round. 

### 2.5. Data Analysis

Survey responses were analysed at the conclusion of each round to establish expert consensus by calculating the percentage of endorsement of each item by panel members. In the absence of a definitive criteria for determining consensus in a Delphi study [45], in this study, items rated as “Essential” or “Important” *and* “Very feasible” or “Feasible” by at least 80% of panel members were classified as endorsed. This cut-off is deemed appropriate given the significant diversity of the experts’ backgrounds and the requirement that both dimensions meet the cut-off for consensus. Items whose consensus ratings on either of the two dimensions fell below 80% were rejected, whereas those with ratings between 70% and 79.9% on both dimensions were re-rated in a subsequent survey round. 

## 3. Results

### 3.1. Characteristics of Panelists

Of the 30 experts invited to take part in the study (as described in Section 2.2), half of them participated. Fifteen additional experts were successfully recruited via a generic invitation distributed to members of the ARACY parent engagement network. Overall, 30 experts completed Round 1, 26 completed Round 2, and 23 completed Round 3 (77% of the Round 1 panel). Of the experts who completed all three survey rounds, the majority of the panelists also reported having at least 5 years of experience in the education (48%) or social services sectors (52%) and 26% had experience in research. Most the experts were between 41 to 60 years old (74%) and identified themselves with the female gender (87%). Although there was representation from all states and territories of Australia, the majority of the experts reported the state of Victoria as their primary place of work (61%).

### 3.2. Statement Ratings

**Figure 1:**
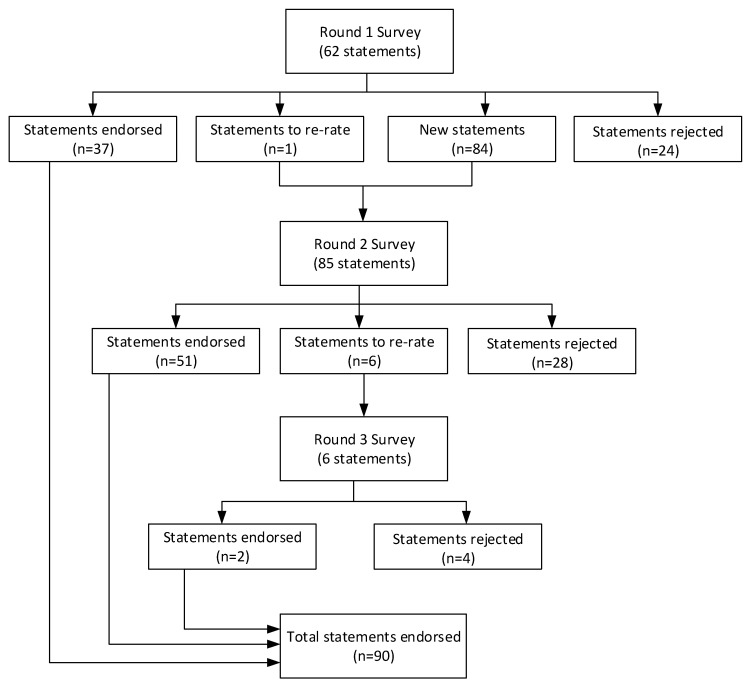
Number of statements that were endorsed, rejected and presented for re-rating at each survey round.

From the literature searches and notes recorded at the workshop described before, 62 statements were presented to the panel for rating in Round 1. Feedback from the experts contributed to 84 new statements and one statement from the previous round that needed to be reappraised in Round 2. In Round 3, six items were presented for re-rating. Figure 1 shows the number of statements in each survey round that were endorsed, rejected or presented for reappraisal in the next round. 

#### 3.2.1. Survey Round 1

In Round 1, 37 statements were endorsed by the panel (≥80% of panel rated statements as important and feasible). Twenty-four statements were rejected (less than 70% of panel members rated statements as important and feasible), and one item required re-rating in Round 2 (between 70% and 79.9% of panel rated statements as important and feasible). The items requiring reappraisal related to selection of programs based on their ability to target risk and protective factors. Eighty-four new statements were created based on comments and suggestions from panel members, including suggestions to revise the wording of two statements for greater clarity. One statement related to program selection and child participation; the other related to program delivery of face-to-face programs. These statements were re-written and presented as new statements for rating in Round 2. See Supplementary file (Table S1) for the full list of strategies that were endorsed, rejected or re-rated in each survey round.

#### 3.2.2. Survey Round 2

Of the 85 statements in Round 2, 51 were endorsed, while 28 were rejected by the panel. Six statements did not attain adequate consensus and were thus presented for re-rating in the final survey in Round 3. Among the statements that were rejected in Round 2, panelists provided relatively low ratings of importance and feasibility on statements related to having teachers facilitate programs targeted at students (22% on importance, 30% on feasibility), using external experts to facilitate programs for parents (39% on importance, 44% on feasibility) and delivering face-to-face programs at home (38% important, 46% feasibility). In addition, there was low consensus on statements about making funds available for schools to support the appointment of teachers to deliver programs (57% on importance, 61% on feasibility) and about schools receiving funding as an incentive to achieve parent engagement targets (50% on importance, 46% on feasibility). Further, a number of statements were rated as important but not feasible by the panel and thus were considered as being rejected. These include statements related to schools receiving funding support for efforts to increase parent engagement targets (88% on importance, 58% on feasibility) and schools selecting programs that include activities in which children can participate (73% on importance, 21% on feasibility). Finally, the statement that was re-rated in this round due to inadequate consensus in the first round did not reach consensus again. This statement was about schools selecting programs based on their ability to target risk and protective factors for the development of child and adolescent mental health problems (81% on importance, 77% on feasibility).

#### 3.2.3. Survey Round 3

In Round 3, six items were presented for reappraisal, of which two reached adequate consensus for endorsement. Across the three rounds, a total of 90 statements were endorsed for both their importance and feasibility as strategies for increasing the uptake of parent education programs in educational settings (see Table 1 for a list of original and new statements that were endorsed and the level of consensus by the panel, arranged in order from the highest to the lowest consensus level obtained on feasibility within each theme and survey round). Responses from the experts indicated that full consensus (100% on both importance and feasibility) was achieved on statements regarding the offering of both universal and targeted programs to meet the diverse needs of parents and children, using a positive tone of voice when promoting programs to parents, and seeking input from parents when developing programs. There was lower agreement in relation to the provision of catering and the use of targeted communication with parents whom school staff believe could benefit most from a program (80% on importance and feasibility), the selection of universal prevention programs with basic strategies for creating positive family communication (80% on importance, 83% on feasibility) and designing programs that focus on the needs of both the family and the school (83% on importance and feasibility). 

## 4. Discussion

This study combined expert consensus and practices to identify and develop a set of strategies that various stakeholders with shared interests and responsibilities in child mental health can take to increase the uptake of parent education programs in early learning and school settings. Despite the wealth of best practices and models for enhancing parent engagement and family–school partnerships in educational settings, there is limited evidence supporting the effectiveness of these well-intended recommendations in improving child mental health and wellbeing. As evident from reviews and meta-analytic work conducted on the topic [12,26,35,36], many recommendations are based on research evidence on educational and/or behavioural outcomes or with reference to particular learnings identified from parent education programs delivered to families, which as mentioned previously are usually not described in terms of actionable strategies for educators and practitioners. Where existing theories and models of engagement and partnerships were conceived primarily by scholars and policy makers [24,25,27,50], the strategies developed in this study were synthesised through an iterative process with an expert panel of researchers, educators and other practitioners.

We found that a group of Australia based educators, service providers and researchers, highly experienced in working with parents and families in educational settings, were able to reach consensus around a broad set of guidelines for increasing parent engagement and uptake of parent education programs in school and preschool settings. Overall, ninety statements that were endorsed by at least 80% of the experts cover strategies that correspond to ten distinct themes: (i) parent education program selection, (ii) increasing the accessibility of parent education programs, (iii) schools’ role in parent education program promotion, (iv) school staff’s role in parent education program promotion, (v) program developers’ role in parent education program promotion, (vi) service providers’ role in parent education program promotion, (vii) increasing the understanding of educational staff on parent engagement and child mental health, (viii) parent education program development, (ix) program delivery and (x) increasing parent and community engagement.

Across the expert panel, the highest consensus was reached for the sets of strategies related to the role of school staff and program developers in program promotion and the set of strategies related to program development. On average, strategies delineated in these three themes were endorsed as both important and feasible by over 92% of the panel. Relatively high consensus was also obtained on the set of strategies related to increasing the understanding of educational staff on parent engagement and child mental health (96% on importance, 89% on feasibility) and the role of the school leadership in increasing parent and community engagement (importance 96%, feasibility 86%). In line with the factors reported by program developers [42] and experts in implementation research in schools [51], the majority of the experts identified strategies about developers’ and providers’ capacity to promote and disseminate their programs to schools and the importance of ensuring the buy-in and support of school leadership and the student wellbeing team as instrumental to the uptake of parent education programs in educational settings.

The experts in the current Delphi study also perceived school leaders/administrators as crucial to developing a school culture that facilitates parent/family engagement, establishing goals and strategies and driving the schools’ efforts at building partnerships with families. This is broadly consistent with a scoping review by Webster et al. [52] which revealed professionals’ recommendations for school administrators to work with teachers and community stakeholders to build relationships and to lead or co-lead initiatives in school health promotion programs. In addition, experts in this study also endorsed the strategies for school leaders to participate in training for a better understanding of the relationship between parenting, child’s mental health and academic performance. This finding supports Webster and colleagues’ recommendations for administrators to attend and participate in professional development training and workshops related to school-based health promotion [52]. That the experts highly ranked the importance and feasibility of engaging parents in co-designing programs was also supported by the literature on implementation science for mental health interventions [51,53,54].

Several statements that were initially derived from the literature [35] failed to attain adequate expert consensus to be included in the final list of endorsed strategies. The majority of the panel agreed that training staff in educational settings to deliver programs (importance 40%, feasibility 43%), and involving teachers in facilitating programs targeted at students (importance 22%, feasibility 30%) were not sufficiently important and feasible as strategies to increase parent uptake of parent education programs in educational settings. Relatedly, there was low consensus among the experts with regard to making funds available for schools to support the appointment of teachers to deliver programs (importance 57%, feasibility 61%), and for schools receiving funding as an incentive to achieve parent engagement targets (importance 50%, feasibility 46%). These findings are somewhat consonant with Cook et al. [51] where experts deemed financial strategies as generally inappropriate for use in schools due to school policies and collective bargaining arrangements. When taken together, these findings show that while experts recognised the benefits of teachers receiving professional development on child mental health (93% on importance, 83% on feasibility) and how to engage parents (importance 93%, feasibility 90%), it is possible that they are also deeply aware of other competing priorities, roles and logistical barriers that teachers face, which would prevent them from effective program delivery.

On the other hand, ratings from the experts suggest that there is a belief that parent education programs should not be facilitated solely by external experts, with a preference for qualified experts being appointed as school staff to do this. Specifically, most experts on the panel supported the notion that schools should recruit suitably qualified staff to build parents’ capacity to engage as partners with the school (importance 100%, feasibility 87%) and that schools should recruit suitably qualified staff to support teacher professional development in engagement with families as partners with the school to improve student mental health and wellbeing outcomes (important 91%, feasibility 83%).

A number of other findings also warrant discussion. First, an examination of the experts’ comments indicates that experts are cognisant of the lack of evidence on strategies to enhance partnerships for supporting student mental health and wellbeing. This is evident from comments received in Round 1 where a number of experts called for statements on developing the evidence base on what works as a strategy for building parent engagement and partnerships, as well as statements on the use of evidence-based programs to increase parent engagement and to improve students’ outcomes. Given the limited research, it is likely that experts drew on their own experience working with educators, families or other service providers in rating the importance and feasibility of the strategies. Second, although experts recognised that it is important for schools to select programs that target modifiable risk factors, they also believed that it is not a feasible strategy for increasing parent uptake of parent education programs (importance 80%, feasibility 57%). Similarly, experts did not reach an adequate level of consensus regarding the feasibility for schools to select programs based on the programs’ ability to target risk and protective factors for the development of child and adolescent mental health problems (73–81% importance, feasibility 70–77% over two survey rounds). These findings may be surprising given the expectation that experts would place higher emphasis on the importance for programs to be targeting modifiable risk factors. One plausible explanation is that some experts may be in favour of schools choosing programs that give consideration to the local community’s unique profile of risk and protective factors over and above programs that target a broad range of modifiable risk factors. Some support for this explanation comes from the high consensus ratings obtained on statements related to schools selecting programs that “demonstrate cultural awareness”, programs that are the “most relevant to the school’s parent population” or are “culturally relevant to the school’s population”. Third, the experts as a group did not deem the strategy of schools selecting online or technology-assisted programs as important and feasible for increasing parent engagement and uptake of parent education programs (this statement was rejected in Round 1 where only 40% and 73% of the panel rated it as important and feasible, respectively). This is an interesting finding given the coincidence that data collection for this study took place during the COVID-19 pandemic in 2020 where in Australia parents, educators and learners were put under pressure to adapt quickly to the online learning environment and to develop innovative ways to exchange information and provide support remotely. In the broader literature on parenting interventions, digital interventions have emerged as alternatives or adjuncts to therapist-guided programs with its purported advantages of increased accessibility, convenience and anonymity, and therefore may overcome some of the barriers experienced by schools and practitioners in implementing face-to-face programs [55,56]. Parents themselves have indicated a preference for online parenting programs to support their child’s mental health [57,58]. There is also preliminary evidence to suggest that technology enhancements could facilitate interactivity and therefore increase engagement and outcomes of interventions [59,60]. That most experts did not think that schools should select online or technology-assisted programs as a strategy to increase parent engagement may reflect an underlying concern that a reliance on technology to build and/or maintain parent–teacher connections may further alienate some families with limited resources or technology literacy. Alternatively, it may reflect some reservations held about parents’ willingness to participate in parent education programs remotely when children return to school and the pressure to lead their child’s learning at home wanes.

This study has several limitations that should be considered. Although comprehensive efforts were made to search relevant English publications identified from Clancy et al.’s review [35] and an update search of the grey literature was conducted three months prior to survey content development, it is by no means an exhaustive and inclusive procedure. Future studies should extend search procedures to include literature in languages other than English and to rerun searches for all relevant academic databases. In addition, we approached only expert participants based in Australia. Although a diverse group of professionals was represented (e.g., educators, researchers, family service providers), being restricted to a local sample meant that the strategies identified from this study may not generalise to countries with very different education and public health systems. We suggest that countries with a different educational system may find the Delphi consensus method with their local experts a useful technique for determining strategies relevant to their contexts. As the focus of this study was on program uptake, whether the strategies for increasing parent uptake would also be useful for increasing parent retention in parent education programs have not been specifically considered in the study. To maximise the accuracy and currency of the strategy statements presented in the surveys, future Delphi studies could also consider seeking the direct inputs of key experts whose work have been drawn upon to develop the statements in the initial survey, to test a draft version of the survey or to participate as an expert panelist on the Delphi study. A logical next step would be to involve policy makers and practitioners (program implementors) in fine-tuning and framing the expert-endorsed recommendations in the language that is familiar and acceptable to these groups of stakeholders. There is also more work to be carried out to bridge the gap in research and practice, such as experimentally testing the recommendations.

Findings from this study can underpin dedicated lines of inquiry on school staff training and preparedness for parent and community engagement, program designer and facilitator preparedness for co-design work with parents, as well as the capacity to disseminate and market their programs and garner stakeholder support. Although the focus of this Delphi study was to identify the strategies to increase the uptake of parent education programs in educational settings with the ultimate aim of improving mental health and wellbeing of children and adolescents, and detailed recommendations for the conduct of family–school partnership interventions are outside its scope, we suggest that in choosing what type of intervention or strategies to adopt to improve parent engagement, one needs to pay attention to the education planning priorities and the local needs of the school community. In order to move forward in this line of work, researchers and program evaluators should strive for better reporting of the forms of parent engagement and partnership building strategies employed in their projects and intervention studies. Further research is essential to advancing the evidence base behind the recommended strategies so as to develop effective practice guidance for the promotion of mental health and wellbeing in children and adolescents. Finally, parent education programs have the potential to empower parents with strategies to support the wellbeing of their children and prevent other difficulties that may arise in stressful circumstances. In health emergency situations such as a pandemic, parents are the key resource for their children during prolonged periods of home confinement and isolation. Whilst it is encouraging to see the innovative ways that educators, program developers and service providers have employed to reach out to parents during the pandemic, there is a need for continued investments by all stakeholders to sustain these efforts and to promote actions for improving the uptake of parent education programs in educational settings.

## 5. Conclusions

The current study aimed to identify strategies for increasing the uptake of parent education programs in preschool and school settings with the aim of promoting child and adolescent mental health and wellbeing through family–school partnerships. Although a number of strategies identified from this study are routinely considered by schools, service providers and program developers, some strategies are overdue for specific targeting and evaluation (e.g., priorities for parent engagement in schools, professional training for educational staff on parent engagement and engagement of parents in co-designing parent education programs). The identified strategies can be promoted as a set of broad, expert-informed recommendations to parent advocates, educators, family service providers, program developers and policy makers, at least some of which will be applicable and of interest to each stakeholder in the public health, social or education sectors.

## Figures and Tables

**Table 1:** List of original and new strategies endorsed by panel across three survey rounds.

Theme/Strategy Endorsed by Panelists	Consensus Level on Importance ^a^	Consensus Level on Feasibility ^a^	Round Where Item Was Endorsed
**Parent education programs selection (8 original + 8 new)**			
Schools should …			
1. select programs that are most relevant to the school’s parent population.	86.7	93.3	1
2. select programs that are culturally relevant to the school’s population.	96.7	90.0	1
3. select programs that treat parents as equal partners.	90.0	90.0	1
4. select programs that include strategies with a research evidence base for being effective.	90.0	90.0	1
5. include parent committees in the process of selecting programs.	86.7	86.7	1
6. select programs that uses a whole school approach.	83.3	86.7	1
7. select universal prevention programs with basic strategies for creating positive family communication.	80.0	83.3	1
8. select programs that can be tailored to parents’ individual needs.	93.3	80.0	1
9. offer both universal and targeted programs in order to meet the diverse needs of parents and children.	100.0	100.0	2
10. select programs that demonstrate cultural awareness.	100.0	96.2	2
11. adapt programs to suit the unique needs and resources of their school communities.	100.0	84.6	2
12. involve the broader parent community, besides parent committees, in the selection of programs.	84.6	84.6	2
13. select programs with a clear evidence base for improving student outcomes.	88.5	84.6	2
14. select programs with evidence on motivating behaviour change in parents (and not just based on appraisals of the program’s “likeability”).	84.6	80.8	2
15. select programs with a universal whole school approach which simultaneously targets teachers, parents and students.	84.6	80.8	2
16. select programs that could be run in multiple rounds each year to allow for “refresher” sessions.	82.6	91.3	3
*Mean of consensus levels for items in this theme*	*89.5*	*87.7*	*-*
**Increasing the accessibility of parent education programs (3 original + 3 new)**			
Program developers should provide parents with choice on how the program is delivered, including			
1. face-to-face options.	93.3	96.7	1
2. flexible timing.	96.7	86.7	1
3. being delivered in community areas near public transport options.	91.7	87.5	2
4. being delivered in non-school venues if the program is face-to-face.	91.3	91.3	3
Programs should provide practical support, including:			
5. catering.	80.0	80.0	1
6. translation and interpreting services.	95.8	87.5	2
*Mean of consensus levels for items in this theme*	*91.5*	*88.3*	*-*
**Parent education program promotion: Schools (6 original + 3 new)**			
Schools should …			
1. promote programs through universal communication strategies to all parents.	90.0	96.7	1
2. use online and e-mediated forums to promote programs.	90.0	93.3	1
3. include parent committees in the promotion of programs that the school is involved in.	90.0	83.3	1
4. use regular social events to promote programs.	86.7	83.3	1
5. create a leadership role so that a member of staff takes responsibility for informing other teachers.	86.7	80.0	1
6. promote programs through targeted communication with the parents whom staff believe could benefit most from a program.	80.0	80.0	1
7. provide programs as a suite of services and supports which families can access and engage with as part of a whole school community hub approach.	91.7	87.5	2
8. appoint skilled staff/professionals in the school to build relationships with families and service providers.	91.7	83.3	2
9. appoint skilled staff/professionals in the school to negotiate the delivery of local services and supports which meet family needs.	91.7	83.3	2
*Mean of consensus levels for items in this theme*	*88.7*	*85.6*	*-*
**Parent education program promotion: School staff (3 original + 6 new)**			
School staff should …			
1. ensure that their tone is positive when approaching parents.	100.0	100.0	1
2. ensure language used when discussing programs with parents is suited to the target demographic.	100.0	96.7	1
3. emphasise potential benefits for the child.	96.7	96.7	1
4. use language that is inclusive, blame-free and shame-free when discussing programs with parents.	100.0	95.8	2
5. advertise programs using inclusive and non-stigmatising language.	100.0	95.8	2
6. use language and a tone of voice that reflects empathy with parents’ frustrations/challenges.	95.8	95.8	2
7. use a strength-based approach when discussing programs with parents.	95.8	95.8	2
8. promote programs that are sensitive to the needs of the family as a whole, rather than focusing only on the child’s school attendance and behaviour.	95.8	87.5	2
9. promote programs that are sensitive to both the needs of the family and the school.	91.7	87.5	2
*Mean of consensus levels for items in this theme*	*97.3*	*94.6*	*-*
**Parent education program promotion: Program developers (8 original + 4 new)**			
Program developers should …			
1. create promotional materials that provide clear details on how to sign up.	96.7	96.7	1
2. create promotional materials that use positive language.	90.0	96.7	1
3. provide schools with publicity materials for their program.	90.0	96.7	1
4. create promotional materials that clearly advertise the practical assistance provided by organisers.	86.7	96.7	1
5. seek the input of school staff for strategies to promote their programs.	93.3	93.3	1
6. create promotional materials that provide clear details on what is required to participate in the program.	90.0	93.3	1
7. provide schools with instructions on how to refer a parent to their program.	90.0	93.3	1
8. meet with school staff to agree upon recruitment processes.	86.7	80.0	1
9. advertise programs using inclusive and non-stigmatising language.	100.0	95.8	2
10. ensure the buy-in of the school leadership and student wellbeing teams prior to promoting the program.	100.0	91.7	2
11. create promotional materials in languages other than English.	91.7	91.7	2
12. offer a suite of communication tools that can be easily adapted for use by an individual school or early childhood centre.	91.7	87.5	2
*Mean of consensus levels for items in this theme*	*92.2*	*92.8*	*-*
**Parent education program promotion: Service providers (all new)**			
Service providers should …			
1. advertise programs using inclusive and non-stigmatising language.	95.8	95.8	2
2. ensure the buy-in of the school leadership and student wellbeing teams prior to promoting the program.	95.8	91.7	2
3. be aware of other programs available at the school.	91.7	87.5	2
4. offer a suite of communication tools that can be easily adapted for use by an individual school or early childhood centre.	87.5	83.3	2
*Mean of consensus levels for items in this theme*	*92.7*	*89.6*	*-*
**Increasing the understanding of educational staff (9 original, 7 new)**			
There should be …			
1. efforts to ensure the reception staff are aware of the programs the school is offering.	100.0	93.3	1
2. guidelines on the language and terms to use when promoting programs to parents.	93.3	93.3	1
3. school policies that emphasise the importance of family engagement.	100.0	90.0	1
4. guidelines for school staff on how to engage parents in programs.	96.7	90.0	1
5. guidelines for teachers on how to approach parents who may feel a sense of shame if invited to a program.	96.7	90.0	1
6. training for school staff regarding student mental health and wellbeing.	96.7	90.0	1
7. professional development for teachers focused on how to engage parents.	93.3	90.0	1
8. training for teachers to see the relationship between parenting, the child’s mental health and their academic performance.	93.3	83.3	1
9. training for principals to see the relationship between parenting, the child’s mental health and their academic performance.	93.3	80.0	1
10. school leadership to ensure the success of programs implemented for the school community.	95.8	95.8	2
11. vision and mission statements in schools/early learning centres that emphasise the importance of students’ mental and physical wellbeing.	91.7	95.8	2
12. professional development for educational staff on the value of parent–teacher/family–school partnership in supporting a child.	100.0	91.7	2
13. opportunities for educational staff to be partners in program implementation and delivery so that they learn through doing.	100.0	87.5	2
14. training for school staff to effect positive change in attitudes towards parent engagement.	100.0	87.5	2
15. professional development for educational staff on the value of parent education programs.	95.8	87.5	2
16. training for front-line administrative/reception staff on how to engage parents.	100.0	83.3	2
*Mean of consensus levels for items in this theme*	*96.7*	*89.3*	*-*
**Parent education program development (all new)**			
Program developers should…			
1. seek input from parents when developing their programs.	100.0	100.0	2
2. seek input from family service providers when developing their programs.	87.5	100.0	2
3. develop an evidence base on what works for family engagement.	100.0	95.8	2
4. develop an evidence base on effective strategies for family–school partnerships.	95.8	91.7	2
5. engage parents in co-designing programs.	95.8	91.7	2
6. design a program based on principles and values that reflect the role of parents in moulding the future of their children.	83.3	83.3	2
7. design programs that focus on the needs of both the family and the school.	83.3	83.3	2
*Mean of consensus levels for items in this theme*	*92.3*	*92.3*	-
**Program Delivery (all new)**			
1. school staff should be offered some training or support if necessary, by experts in the relevant subject area.	100.0	87.0	2
2. schools/service providers should incorporate social elements when running programs for families (e.g., parents are able to share food and meet with each other).	95.7	82.6	2
*Mean of consensus levels for items in this theme*	*97.8*	*84.8*	*-*
**Increasing parent and community engagement (all new)**			
School leadership is required to …			
1. develop a school culture that enables parent/family engagement.	100.0	91.3	2
2. establish goals and strategies for parent/family engagement.	100.0	91.3	2
3. drive schools’ efforts at building partnerships with families.	95.7	87.0	2
Schools should …			
4. recruit suitably qualified staff to build parents’ capacity to engage as partners with the school to improve student mental health and wellbeing outcomes.	100.0	87.0	2
5. use evidence-based school improvement strategies to improve student mental health and wellbeing outcomes.	91.3	87.0	2
6. use evidence-based strategies to improve partnerships with community groups with shared interests in child and family wellbeing.	95.7	82.6	2
7. use evidence-based school improvement strategies to improve the tripartite partnership between school, family and community groups with shared interests in child and family wellbeing.	95.7	82.6	2
8. recruit suitably qualified staff to support teacher professional development in engagement with families as partners with the school to improve student mental health and wellbeing outcomes.	91.3	82.6	2
9. recruit suitably qualified staff to engage with community groups with shared interests in child and family wellbeing.	91.3	82.6	2
*Mean of consensus levels for items in this theme*	*95.7*	*86.0*	*-*

Note. Thematic headings are in bold. ^a^ Round 1 panel (*n* = 30), Round 2 panel (*n* = 26), Round 3 panel (*n* = 23–26). Round 3 survey was partially completed by three panelists and fully completed by 23 panelists.

## Data Availability

De-identified data for this study are available upon reasonable request from the corresponding author.

## Supplementary Materials

The following are available online at https://www.mdpi.com/article/10.3390/ijerph18073524/s1, Table S1: Items endorsed, rejected or re-rated at each survey round.
