# Supplementary material for: Strategies to Increase Uptake of Parent Education Programs in Preschool and School Settings to Improve Child Outcomes: A Delphi Study
Source: Int J Environ Res Public Health. 2021 Mar 29;18(7):3524. doi: 10.3390/ijerph18073524 (PMC8036819; doi:10.3390/ijerph18073524)
Supplement: Supplementary file 1 [file ijerph-18-03524-s001.pdf]

Supplementary Table S1: Items endorsed, rejected or re-rated in each survey round

Article: *Strategies to Increase Uptake of Parent Education Programs in Preschool and School Settings to Improve Child Outcomes: A Delphi Study*

Round 1 Survey

Endorsed (≥80% expert consensus)	To be re-rated (70-79.9% expert consensus)	Rejected (<70% expert consensus)
**Parent education programs selection**<br><br>***Schools should…***<br>1. select programs that are most relevant to the school's parent population.<br>2. include parent committees in the process of selecting programs.<br>3. select programs that uses a whole school approach.<br>4. select programs that are culturally relevant to the school’s population.<br>5. select programs that can be tailored to parents’ individual needs.<br>6. select programs that treat parents as equal partners.<br>7. select programs that include strategies with a research evidence base for being effective.<br>8. select universal prevention programs with basic strategies for creating positive family communication.	***Schools should…***<br>1. select programs based on their ability to target risk and protective factors for the development of child and adolescent mental health problems.	***Schools should…***<br>1. select programs that target modifiable risk factors (risk factors that can be addressed through actions).<br>2. select programs that include components for children.*<br>3. schools should include children in the recruitment process.<br>4. select programs that are not run by organisations associated with child protective services.<br>5. select online or technology assisted programs.<br>6. select prevention programs with basic information that is beneficial to all parents.<br>7. select universal prevention programs with basic strategies for creating reasonable expectations for their child’s behaviour.<br>8. select universal prevention programs with basic strategies for creating consistent expectations for their child’s behaviour.<br>9. select universal prevention programs with basic strategies for creating regular family communication.

Endorsed (≥80% expert consensus)	To be re-rated (70-79.9% expert consensus)	Rejected (<70% expert consensus)
		10. select universal prevention programs with basic strategies for engaging with children and adolescents.<br>11. provide programs that are translated to languages other than english.<br><br>**re-written as new statement for rating in Round 2.*
**Program development**<br><br>Nil.	Nil.	***program developers should…***<br>1. make efforts to reduce potential stigma in their program design.<br>2. seek the input of educators when developing their programs.<br>3. seek the input of school staff when developing their programs.
**Increasing the accessibility of parent education programs**<br><br>***Program developers should provide parents with choice on how the program is delivered, including…***<br>1. face-to-face options<br>2. flexible timing.<br>***The programs should provide practical support, including…***<br>1. catering.	Nil.	***Program developers should provide parents with choice on how the program is delivered, including…***<br>1. online options.<br>2. flexible locations.<br>3. transportation assistance.<br>***The programs should provide practical support, including…***<br>1. child minding.<br>2. be delivered at school if the program is face-to-face.*<br><br>**re-written as new statement for rating in Round 2.*

Endorsed (≥80% expert consensus)	To be re-rated (70-79.9% expert consensus)	Rejected (<70% expert consensus)
**Parent education program promotion**<br><br>***Schools should…***<br>1. promote programs through universal communication strategies to all parents.<br>2. promote programs through targeted communication with the parents whom staff believe could benefit most from a program.<br>3. include parent committees in the promotion of programs that the school is involved in.<br>4. create a leadership role so that a member of staff takes responsibility for informing other teachers.<br>5. use regular social events to promote programs.<br>6. use online and e-mediated forums to promote programs.<br>***School staff should…***<br>1. ensure language used when discussing programs with parents is suited to the target demographic.<br>2. emphasise potential benefits for the child.<br>3. ensure that their tone is positive when approaching parents.<br>***Program developers…***<br>1. seek the input of school staff for strategies to promote their programs.<br>2. create promotional materials that use positive language.<br>3. create promotional materials that clearly advertise the practical assistance provided by organisers.<br>4. create promotional materials that provide clear details on how to sign up.	Nil.	***Schools should…***<br>1. invite parents to initial trials of programs.<br>***Program developers should…***<br>1. provide financial incentives for participating.<br>2. create promotional materials that clearly advertise incentives for participating.

Endorsed (≥80% expert consensus)	To be re-rated (70-79.9% expert consensus)	Rejected (<70% expert consensus)
5. create promotional materials that provide clear details on what is required to participate in the program.<br>6. meet with school staff to agree upon recruitment processes.<br>7. provide schools with instructions on how to refer a parent to their program.<br>8. provide schools with publicity materials for their program.		
**Increasing the understanding of educational staff**<br><br>***There should be…***<br>1. guidelines for school staff on how to engage parents in programs.<br>2. guidelines for teachers on how to approach parents who may feel a sense of shame if invited to a program.<br>3. guidelines on the language and terms to use when promoting programs to parents.<br>4. professional development for teachers focused on how to engage parents.<br>5. training for school staff regarding student mental health and wellbeing.<br>6. training for teachers to see the relationship between parenting, the child's mental health, and their academic performance.<br>7. training for principals to see the relationship between parenting, the child's mental health, and their academic performance.<br>8. efforts to ensure the reception staff are aware of the programs the school is offering.	Nil.	Nil.

Endorsed (≥80% expert consensus)	To be re-rated (70-79.9% expert consensus)	Rejected (<70% expert consensus)
9. school policies that emphasise the importance of family engagement.		
**Increasing teachers' involvement in parent education programs**<br><br>Nil.	Nil.	1. Staff in educational settings should be trained to deliver programs.<br>2. Teachers should participate in school facilitated programs.

Round 2 Survey

Endorsed (≥80% expert consensus)	To be re-rated (>70<79% expert consensus)	Rejected (<70% expert consensus)
**Parent education programs selection**<br><br>***Schools should…***<br>1. select programs with evidence on motivating behaviour change in parents (and not just based on appraisals of the program's 'likeability').<br>2. select programs with a clear evidence base for improving student outcomes.<br>3. adapt programs to suit the unique needs and resources of their school communities.<br>4. offer both universal and targeted programs in order to meet the diverse needs of parents and children.<br>5. involve the broader parent community, besides parent committees, in the selection of programs.	***Schools should…***<br>1. select programs that could be run in multiple rounds each year to allow for 'refresher' sessions.	***Schools should…***<br>1. receive funding support for efforts to increase parent engagement.<br>2. receive funding as an incentive to achieve parent engagement targets.<br>3. select programs that could be run in multiple rounds each year to help reduce the stigma of participating in these programs.<br>4. select programs that include activities in which children can participate.

Endorsed (≥80% expert consensus)	To be re-rated (>70<79% expert consensus)	Rejected (<70% expert consensus)
6. select programs that demonstrate cultural awareness.<br>7. select programs with a universal whole school approach which simultaneously targets teachers, parents and students.		
**Program development**<br><br>***Program developers should…***<br>1. develop an evidence base on effective strategies for family-school partnerships.<br>2. develop an evidence base on what works for family engagement.<br>3. design a program based on principles and values that reflect the role of parents in moulding the future of their children.<br>4. engage parents in co-designing programs.<br>5. seek input from family service providers when developing their programs.<br>6. seek input from parents when developing their programs.<br>7. design programs that focus on the needs of both the family and the school.	***Program developers should…***<br>1. engage students where possible in co-designing programs.<br>2. incorporate current school protocols (e.g. managing challenging behaviours, school values) and use existing strengths of the school, if a whole school approach is used.	***Program developers should…***<br>1. develop an evidence base on effective strategies for engaging community groups of people with shared interests on child and family wellbeing.<br>2. engage educators in co-designing programs.<br>3. seek input from educators with joint expertise in the areas of health, mental health and wellbeing, disability and inclusion.
**Increasing the accessibility of parent education programs**<br><br>***Program developers should provide parents with choice on how the program is delivered, including…***<br>1. being delivered in community areas near public transport options.	***Program developers should provide parents with choice on how the program is delivered, including…***	***Program developers should provide parents with choice on how the program is delivered, including…***<br>1. being delivered at home if the program is face-to-face.

Endorsed (≥80% expert consensus)	To be re-rated (>70<79% expert consensus)	Rejected (<70% expert consensus)
The programs should provide practical support, including…<br>1. translation and interpreting services.	1. being delivered in non-school venues if the program is face-to-face.<br>***The programs should provide practical support, including…***<br>1. being delivered at the early childhood centre or school if the program is face-to-face.	2. being delivered via a combination of face-to-face and online modalities.
**Parent education program promotion**<br><br>***Schools should…***<br>1. provide programs as a suite of services and supports which families can access and engage with as part of a whole school community hub approach.<br>2. appoint skilled staff/professionals in the school to build relationships with families and service providers.<br>3. appoint skilled staff/professionals in the school to negotiate the delivery of local services and supports which meet family needs.<br>***School staff should…***<br>1. promote programs that are sensitive to the needs of the family as a whole, rather than focusing only on the child's school attendance and behaviour.<br>2. promote programs that are sensitive to both the needs of the family and the school.	Nil.	***Schools should…***<br>1. engage community/religious groups to support the programs and help to draw parents to the programs.<br>2. engage parents who are willing to lead program promotion activities during social events and activities.<br>***School staff should…***<br>1. lead program promotion activities during social events and activities for families.<br>***Program developers should…***<br>1. highlight the relevance of the program in meeting the needs of the school.<br>***Service providers should…***<br>1. provide programs as a suite of services and supports which families can access and engage with as part of a whole school community hub approach.

Endorsed (≥80% expert consensus)	To be re-rated (>70<79% expert consensus)	Rejected (<70% expert consensus)
3. use a strength-based approach when discussing programs with parents.<br>4. use language that is inclusive, blame-free and shame-free when discussing programs with parents.<br>5. use language and a tone of voice that reflects empathy with parents' frustrations/challenges.<br>6. advertise programs using inclusive and non-stigmatising language.<br>***Program developers should…***<br>1. create promotional materials in languages other than English.<br>2. offer a suite of communication tools that can be easily adapted for use by an individual school or early childhood centre.<br>3. advertise programs using inclusive and non-stigmatising language.<br>4. ensure the buy-in of the school leadership and student wellbeing teams prior to promoting the program.<br>***Service providers should…***<br>1. offer a suite of communication tools that can be easily adapted for use by an individual school or early childhood centre.<br>2. be aware of other programs available at the school.<br>3. advertise programs using inclusive and non-stigmatising language.<br>4. ensure the buy-in of the school leadership and student wellbeing teams prior to promoting the program.		2. highlight the relevance of the program in meeting the needs of the school.<br>3. have a good understanding of the mental health issues at the school.

Endorsed (≥80% expert consensus)	To be re-rated (>70<79% expert consensus)	Rejected (<70% expert consensus)
**Increasing the understanding of educational staff**<br><br>***There should be…***<br>1. professional development for educational staff on the value of parent-teacher/family-school partnership in supporting a child.<br>2. professional development for educational staff on the value of parent-education programs.<br>3. opportunities for educational staff to be partners in program implementation and delivery, so they learn through doing.<br>4. training for school staff to effect positive change in attitudes towards parent engagement.<br>5. training for front-line administrative/reception staff on how to engage parents.<br>6. vision and mission statements in schools/early learning centres that emphasise the importance of students' mental and physical wellbeing.<br>7. school leadership to ensure the success of programs implemented for the school community.	Nil.	***There should be…***<br>1. professional development for all levels of educational staff on state policies and terms of reference on family-school partnerships.
**Program delivery**<br><br>1. School staff should be offered some training or support if necessary, by experts in the relevant subject area.<br>2. Schools/service providers should incorporate social elements when running programs for families (e.g. parents are able to share food and meet with each other).	1. Programs should be co-facilitated by school staff and external staff so that relationships built between families and school staff can be	1. Teachers should be involved in program delivery as part of a school-wide family engagement and student wellbeing approach so that it is effective and sustainable.<br>2. Funding should be available to schools to support the appointment of teachers to deliver the programs.

Endorsed (≥80% expert consensus)	To be re-rated (>70<79% expert consensus)	Rejected (<70% expert consensus)
	sustained throughout their child's schooling after the programs have ended.	3. Programs should be facilitated by school staff if their involvement would generate greater trust from families.<br>4. Programs should be co-facilitated by school staff and external staff so that relationships built between families and school staff can be sustained throughout their child's schooling after the programs have ended.<br>5. Programs should be facilitated by external staff or service providers as they can offer a new perspective.<br>6. Teachers need to build relationships and trust with parents before they undertake a program together.<br>7. There should be a teacher-parent relationship that focusses on a partnership to support children's learning and wellbeing.<br>8. Programs should be facilitated by teachers if the programs are targeted at students.<br>9. Programs should be facilitated by external experts if the programs are targeted at parents.<br>10. Program developers/service providers should provide supervision for teachers seeking to further their skills in delivering the programs.<br>11. Program developers/service providers should provide drop-in or scheduled appointments for parents seeking further discussion.
**Increasing parent and community engagement**<br><br>***Schools should…***	Nil.	Nil.

Endorsed (≥80% expert consensus)	To be re-rated (>70<79% expert consensus)	Rejected (<70% expert consensus)
1. recruit suitably qualified staff to engage with community groups with shared interests in child and family wellbeing.<br>2. recruit suitably qualified staff to build parents' capacity to engage as partners with the school to improve student mental health and wellbeing outcomes.<br>3. recruit suitably qualified staff to support teacher professional development in engagement with families as partners with the school to improve student mental health and wellbeing outcomes.<br>4. use evidence-based school improvement strategies to improve student mental health and wellbeing outcomes.<br>5. use evidence-based strategies to improve partnerships with community groups with shared interests in child and family wellbeing.<br>6. use evidence-based school improvement strategies to improve the tripartite partnership between school, family and community groups with shared interests in child and family wellbeing.<br>***School leadership should…***<br>1. drive schools' efforts at building partnerships with families.<br>2. develop a school culture that enables parent/family engagement.<br>3. establish goals and strategies for parent/family engagement.		

Round 3 Survey

Endorsed (≥80% expert consensus)	Rejected (<70% expert consensus)
**Parent education programs selection**<br><br>***Schools should…***<br>1. select programs that could be run in multiple rounds each year to allow for 'refresher' sessions.	Nil.
**Program development**<br><br>Nil.	***Program developers should****…*<br>1. engage students where possible in co-designing programs.<br>2. incorporate current school protocols (e.g., managing challenging behaviours, school values) and use existing strengths of the school, if a whole school approach is used.
**Increasing the accessibility of parent-education programs**<br><br>***Program developers should provide parents with choice on how the program is delivered, including…***<br>1. being delivered in non-school venues if the program is face-to-face.	***The programs should provide practical support, including...***<br>1. being delivered at the early childhood centre or school if the program is face-to-face.
**Program delivery**<br><br>Nil.	1. Programs should be co-facilitated by school staff and external staff so that relationships built between families and school staff can be sustained throughout their child's schooling after the programs have ended.

Note. In the surveys, each statement starts with the stem, "*In order to increase parent engagement in parent-education programs, …*".
